# Cold Exposure Shifts Gut Microbial Butyrate Synthesis Toward the Lysine-Dependent and But-Mediated Terminal Pathways to Enhance Cold Tolerance in Min Pigs

**DOI:** 10.3390/microorganisms14071575

**Published:** 2026-07-19

**Authors:** Yang Chang, Xinlei Liu, Lujing Song, Fei Xu, Ziwen Zhang, Miao Yu, Guandong Wu, Dongjie Zhang, Chunzhu Xu

**Affiliations:** 1 Key Laboratory of Animal Cellular and Genetics Engineering of Heilongjiang Province, College of Life Science, Northeast Agricultural University, Harbin 150030, China; changy051@neau.edu.cn (Y.C.); s2509012002@neau.edu.cn (X.L.); 18911250958@163.com (L.S.); s240901025@neau.edu.cn (F.X.); lisa_i_like@163.com (Z.Z.); s230901001@neau.edu.cn (M.Y.); s230902039@neau.edu.cn (G.W.); 2 Institute of Animal Husbandry, Heilongjiang Academy of Agricultural Sciences, Harbin 150086, China

**Keywords:** Min pigs, gut microbiota, butyrate synthesis, lysine pathway, adipose thermogenesis

## Abstract

This study combined seasonal observation in Min pigs and acute cold challenge experiments in Min pigs and Large White pigs to analyze changes in gut butyrate synthesis under cold exposure and its association with thermogenesis. Compared with summer, Min pigs in winter showed significantly higher Bacteroidota abundance (*p* = 0.006), lysine-pathway genes (*p* < 0.05), and relative gene abundance of the but terminal pathway (*p* < 0.05). Fecal (47.96 vs. 40.24 µmol/L, *p* = 0.020) and serum (4.64 vs. 2.17 µmol/L, *p* = 0.046) butyrate were also elevated and correlated with 10 thermogenesis-related genes (*p* < 0.05). Acute cold challenge increased serum butyrate (*p* = 0.017) and *SLC16A1* expression (adjusted *p* = 0.027) only in Min pigs. Min pigs exhibited higher lysine pathway abundance and greater but terminal contribution than Large White pigs. Metagenomic binning recovered 40 lysine-pathway MAGs (27 unique to Min pigs) and 15 dual-pathway MAGs (11 unique to Min pigs), with Bacteroidota MAGs harboring complete lysine and dual terminal pathways. Collectively, cold exposure correlates with enrichment of lysine-dependent and but terminal butyrate synthesis pathways, highlighting butyrate-producing bacteria as candidate taxa for further investigation of cold-induced gut metabolic remodeling in pigs.

## 1. Introduction

Cold stress disrupts energy metabolism and physiological homeostasis, requiring complex regulatory mechanisms to maintain body temperature and energy balance [1,2,3]. The gut microbiota and its metabolites are key in host cold adaptation [4,5,6]. Short-chain fatty acids (SCFAs), microbial fermentation end-products, serve as energy sources and signaling molecules that regulate metabolic homeostasis and thermogenesis [7,8,9]. Among them, butyrate influences thermogenesis by providing energy and modulating epigenetic and metabolic pathways, affecting thermogenic gene expression in adipose tissue [10,11]. Exogenous butyrate enhances brown adipose tissue activity and energy expenditure in mice, suggesting endogenous production may support cold adaptation [12]. However, how gut microbiota remodels butyrate synthesis under natural cold remains unclear and warrants systematic study.

Butyrate is produced by gut microbiota through four metabolic pathways: the acetyl-CoA, lysine, glutarate, and 4-aminobutyrate pathways. The acetyl-CoA pathway mainly depends on carbohydrate substrates, whereas the lysine pathway uses lysine as its primary amino acid precursor. Despite differences in substrates and intermediate reactions, these pathways converge at terminal butyrate synthesis mediated by either butyryl-CoA:acetate CoA-transferase (but) or butyrate kinase (buk) [13,14]. However, whether the relative contributions of these pathways and terminal reactions adaptively change under cold conditions remains unclear.

Gut microbiota-derived butyrate enters the circulation and is taken up by peripheral tissues via MCT1 and SMCT1, encoded by solute carrier family 16 member 1 (*SLC16A1*) and solute carrier family 5 member 8 (*SLC5A8*), respectively, thereby mediating metabolite transfer between the gut and metabolic tissues [15]. As a key microbial metabolite, butyrate participates in energy metabolism and acts as a signaling molecule regulating adipose tissue function. Thus, the “gut microbiota—butyrate—host metabolism” axis may contribute to cold adaptation. However, systematic comparisons of this axis across host breeds and its functional significance remain limited.

Pigs are key livestock, with local and commercial breeds differing in energy metabolism and cold tolerance [16]. Min pigs, a Chinese indigenous breed from cold regions such as Heilongjiang, exhibit strong cold resistance [17], whereas commercial breeds like Large White pigs rely on temperature-controlled environments and show weaker cold tolerance [18]. Although gut microbiota composition differs markedly between these breeds [19], whether these differences affect butyrate synthesis potential and pathway configuration, contributing to breed-specific cold adaptation, remains unclear due to the lack of systematic functional analysis. Therefore, we hypothesized that long-term cold exposure may have altered the contribution of distinct butyrate synthesis pathways in the gut microbiota of Min pigs, and that Min pigs and Large White pigs differ in both microbial butyrate synthesis potential and host butyrate transporter responses to cold stimulation. To systematically investigate the remodeling of the butyrate synthesis network of the gut microbiota under cold environments, we conducted two complementary experiments using Min pigs and Large White pigs. The seasonal study used adult Min pigs, whose gut microbiota is fully developed and metabolic phenotypes are stable, to capture long-term adaptive remodeling of gut microbial butyrate synthesis under natural cold, analyzing microbial composition, pathway gene abundances, and butyrate concentrations in feces and serum. The acute cold challenge used 2-month-old piglets to simulate a common production scenario in which young piglets are vulnerable to cold stress. The short-term (3-day) cold treatment enabled us to capture immediate breed-specific metabolic responses while excluding long-term environmental confounders, allowing direct comparison of innate cold-response differences between Min pigs and Large White pigs. Additionally, we integrated metagenomic binning to recover metagenome-assembled genomes (MAGs) harboring complete butyrate synthesis pathways, enabling us to pinpoint key taxa contributing to the observed pathway shifts. Given that Large White pigs are not adapted to prolonged cold exposure under natural conditions, the seasonal study was conducted exclusively in Min pigs. Collectively, these investigations aim to clarify the regulatory patterns of gut microbial butyrate synthesis under cold stress and to provide a theoretical foundation for future nutritional strategies to improve energy utilization and cold adaptation in pigs.

## 2. Materials and Methods

### 2.1. Experimental Design and Sample Collection

The seasonal experiment was performed at the Lanxi Min Pig National Conservation Farm (Heilongjiang, China) using 12 female Min pigs (150 days old, 95 kg) from the same closed herd. Animals were housed in group pens (4 × 6 m; 4–5 pigs/pen) with dried corn stalks as bedding, fed a standard corn–soybean diet (digestible energy 12.8 MJ/kg, crude protein 16.2%) twice daily, and provided water ad libitum. Management protocols were standardised year-round with no seasonal diet changes. The same pigs were sampled in both mid-June (summer) and October (winter, non-heating period). Summer mean maximum 28 °C (diurnal variation 6–9 °C, humidity 68%). Winter mean maximum 12 °C (diurnal variation 8–12 °C, humidity 58%). Fecal samples were collected from all 12 pigs per season. For metagenomic sequencing, every 4 pigs were pooled into 3 composite samples. For serum butyrate and adipose transcriptomics, 3 individual pigs were randomly selected per season. Fecal butyrate was measured in 8 individuals per season. Blood was collected from the anterior vena cava. Adipose biopsies were taken from the dorsal cervical region. All samples were snap-frozen and stored at −80 °C.

For the acute cold challenge, twelve 2-month-old piglets (6 Min pigs, 6 Large White pigs; 8–10 kg; females) were housed in temperature-controlled pens (1.5 × 2.0 m; 3 piglets/pen) with rice husk bedding. After 7-day acclimation at 25 °C, piglets were randomly assigned to control (25 °C) or cold (8 °C) for 72 h, yielding four groups (*n* = 3 each). Feed intake and rectal temperature were recorded daily and all groups received identical management without antibiotics or probiotics. After 72 h, piglets were euthanised by intravenous injection of sodium pentobarbital (100 mg/kg). All samples were then collected immediately. Blood was collected from the anterior vena cava before euthanasia. Subcutaneous white adipose tissue (0.5–1 cm depth) at the last rib level was collected in RNAlater^®^ (Invitrogen, Thermo Fisher Scientific, Waltham, MA, USA) within 30 s. Cecal contents were collected for metagenomic analysis. All samples were snap-frozen and stored at −80 °C. All procedures were approved by the Laboratory Animal Ethics Committee of Northeast Agricultural University (Approval No. NEAUEC20230102).

### 2.2. Seasonal Comparison of Gut Microbial Composition and Butyrate Synthesis Genes in Min Pigs

The metagenomic data used for the seasonal experiment in this study were obtained from a previous study [20]. Using these data, we compared gut microbiota composition at the phylum and genus levels between winter and summer. Functional genes were annotated using the KEGG database. To evaluate the relative structure of butyrate synthesis pathways, we normalized the abundances of target genes within each sample by scaling their sum to 1, allowing calculation of each gene’s proportional contribution to the pathway gene set. This normalized value reflects the compositional distribution of functional genes, not direct pathway activity or metabolic flux. We then systematically assessed changes in both the abundance and the normalized abundance ratio of these key genes between winter and summer.

### 2.3. Seasonal Comparison of Butyrate Levels in Serum and Feces of Min Pigs

In the seasonal experiment, serum butyrate concentration was measured using a targeted SCFA method. Serum samples were processed, and 4-methylvaleric acid was added as an internal standard, prior to detection by GC-MS (7890 GC coupled with 5977B MSD, Agilent Technologies, Santa Clara, CA, USA). Chromatographic separation was performed on an Agilent DB-FFAP column (30 m × 250 μm × 0.25 μm) with helium as the carrier gas. Butyrate concentration was quantified using the internal standard method with MSD ChemStation software (vE.02.02.1431).

Fecal butyrate concentration was determined using a commercial ELISA kit (Butyric Acid ELISA Kit, Kehanshengbio, Shanghai, China) according to the manufacturer’s instructions. Fecal samples were homogenized in PBS and centrifuged, and the supernatant was analyzed. The assay performance parameters were as follows: linear regression correlation coefficient (R) ≥ 0.990; minimum detection limit < 1.0 μmol/L; no cross-reactivity with other soluble structural analogues; intra-assay and inter-assay coefficients of variation (CV) both <15%. Butyrate concentration was calculated based on absorbance at 450 nm using a standard curve.

### 2.4. Seasonal Transcriptome Analysis of Subcutaneous Adipose Tissue in Min Pigs

Total RNA was extracted from adipose tissue using TRIzol reagent (Invitrogen, Carlsbad, CA, USA) following the manufacturer’s instructions. RNA purity was assessed by measuring the OD260/280 ratio using a NanoDrop spectrophotometer (Thermo Fisher Scientific, Waltham, MA, USA), and only samples with ratios between 1.8 and 2.2 were used. RNA integrity was evaluated using an Agilent 2100 Bioanalyzer (Agilent Technologies, Santa Clara, CA, USA), with data analyzed by 2100 Expert software (vB.02.10), and samples with RIN ≥ 8.0 were considered acceptable for library construction. Libraries were constructed and sequenced on the Illumina NovaSeq X Plus platform (Illumina, Inc., San Diego, CA, USA). Transcriptome sequencing was performed by Majorbio (Shanghai, China). Raw reads were first quality-controlled using fastp (v0.23.4) [21]. The resulting clean reads were aligned to the reference genome using HISAT2 (v2.1.0) [22]. Transcript assembly was performed with StringTie (v2.1.3) [23], and gene expression levels were quantified using RSEM (v1.3.3) [24] with TPM (Transcripts Per Million) normalization. Differential expression analysis was conducted using DESeq2 (v1.34.0) [25], with thresholds of |log_2_FC| ≥ 2 and adjusted *p* < 0.05. KEGG enrichment analysis was performed using DAVID (v6.8) [26].

### 2.5. Breed Comparison of Serum Butyrate Levels Under Acute Cold Challenge

In the acute cold challenge experiment, serum samples were collected to compare butyrate concentrations between Min pigs and Large White pigs under different temperature conditions. Butyrate was quantified using GC-MS/MS system consisting of an Agilent 7890B gas chromatograph and 7000D triple quadrupole mass spectrometer (Agilent Technologies, Santa Clara, CA, USA). After acidification, samples were extracted with MTBE containing an internal standard. Chromatographic separation was performed on a DB-FFAP capillary column (30 m × 0.25 mm × 0.25 μm), and mass spectrometry was conducted in multiple reaction monitoring (MRM) mode. Butyrate quantification was performed by MetWare Biotechnology Co., Ltd. (Wuhan, China).

### 2.6. Transcriptome Analysis of Adipose Tissue in Min Pigs and Large White Pigs Under Acute Cold Challenge

In the acute cold challenge experiment, subcutaneous adipose tissue samples were collected from Min pigs and Large White pigs. RNA extraction and quality assessment were performed as described in Section 2.4. Libraries were constructed and sequenced on the Illumina NovaSeq X Plus platform (Illumina, Inc., San Diego, CA, USA) to generate 150 bp paired-end reads. Raw sequencing data were quality-controlled using fastp (v0.20.0) [21], and rRNA contamination was removed using Bowtie2 (v2.2.8) [27]. The resulting clean reads were aligned to the pig reference genome Sscrofa11.1 using HISAT2 (v2.1.0) [28]. Transcript reconstruction was performed using StringTie (v1.3.1) [23], and gene expression levels were quantified using RSEM (v1.3.3). Differential expression analysis was conducted using edgeR (v3.42.4) with thresholds of FDR < 0.05 and |log_2_FC| > 2 [29]. Transcriptome sequencing was performed by Gene Denovo Biotechnology Co. (Guangzhou, China). The expression levels of the butyrate transporter genes *SLC16A1* and *SLC5A8* were then obtained from the transcriptome data for comparisons between breeds and between temperature conditions.

### 2.7. Breed Comparison of Butyrate-Producing Bacteria and Functional Genes

In the acute cold challenge, cecal contents from Min pigs and Large White pigs under control and cold conditions were collected for metagenomic sequencing. DNA was extracted, paired-end libraries prepared, and sequencing performed on the Illumina NovaSeq X Plus (Illumina, Inc., San Diego, CA, USA). Reads were quality-checked with fastp (v0.23.0) [21] and host contamination removed with BWA (v0.7.17) [30]. Reads were assembled with MEGAHIT (v1.2.9) [31], genes predicted with Prodigal (v2.6.3) [32], and a non-redundant gene catalogue generated using CD-HIT (v4.6.1) [33]. Gene abundance was quantified with SOAPaligner (v2.21) [34], while taxonomic and functional annotations were performed with DIAMOND (v2.0.13) [35] against the NCBI NR, eggNOG, KEGG, CARD, and VFDB databases. The buk pathway was represented by K00634 and K00929, whereas the but pathway was represented by K01034 and K01035. Among these, K00929, K01034, and K01035 were used as terminal enzyme markers. Principal component analysis (PCA) was used to compare gut microbiota carrying terminal enzyme genes for butyrate synthesis between Min pigs and Large White pigs. Additionally, a terminal enzyme gene set was constructed and normalized within each sample, with the total abundance of target genes standardized to one to calculate the normalized abundance ratio of each gene in the terminal synthesis pathways. Abundance and contribution differences in the but and buk pathways were evaluated, and bacterial contributions to terminal enzyme genes were analyzed.

### 2.8. Screening of Butyrate Synthesis MAGs via Metagenomic Binning

First, contigs from metagenomic assembly were length-filtered, with those ≥1000 bp retained. Binning was performed using MetaBAT (v2.12.1) [36], CONCOCT (v0.5.0) [37], and MaxBin2 (v2.2.5) [38], and the resulting bins were consolidated using DAS_Tools (v1.1.0) [39], followed by refinement with RefineM (v0.0.24) [40]. The completeness and contamination of the MAGs were assessed using CheckM (v1.0.12) [41], and MAGs with medium or higher quality (completeness ≥ 50%, contamination ≤ 10%) were retained. Gene prediction was performed using Prodigal (v2.6.3) [32], taxonomic classification was assigned using GTDB-Tk (v2.3.0, database Release 214), and functional annotation was conducted by aligning sequences against the eggNOG, KEGG, and CARD databases using DIAMOND (v2.0.13, e-value ≤ 1 × 10^−5^). Functional annotation against the CAZy database (v12) was performed using HMMER (v3.1b2). The relative abundance of MAGs in the community was calculated and visualized based on taxonomic annotation and gene abundance.

To ensure analytical accuracy, only MAGs with functionally complete butyrate synthesis pathways were retained. Given that the acetyl-CoA pathways and lysine pathways were predominant in our data, all subsequent analyses focused exclusively on these two core pathways. To identify butyrate producers with a complete acetyl-CoA pathway, we intersected MAGs carrying core acetyl-CoA genes (K00074, K00626, K01715) with those containing terminal buk (K00634, K00929) or but (K01034, K01035) pathway genes. The same intersection approach was applied to MAGs with lysine pathway genes (K01843, K01844, K18011–K18014) to retrieve lysine-dependent butyrate producers. Finally, MAGs encompassing both pathways were combined to define all butyrate-producing taxa harbouring at least one full synthesis pathway. A phylogenetic analysis of 78 butyrate-producing MAGs was performed using the conserved marker genes from the GTDB v214.1 database to construct a phylogenetic tree and analyze the evolutionary relationships between the genomes. To clearly present the functional characteristics of each MAG, information on their butyrate synthesis pathways, terminal pathway and presence or absence across the two pig breeds was integrated into the outer ring of the phylogenetic tree and visualized using Chiplot (https://www.chiplot.online, China).

### 2.9. Statistics and Reproducibility

Group comparisons were performed using Welch’s *t*-tests. Two-tailed Welch’s *t*-tests were applied to analyze gut microbiota composition at the phylum and genus levels, fecal and serum butyrate concentrations in Min pigs across different seasons, serum butyrate levels between pig breeds and under different temperature treatments, as well as the abundance of key genes involved in four butyrate synthesis pathways and three terminal genes. By contrast, one-tailed Welch’s *t*-tests were used to assess seasonal variations in butyrate synthesis genes, to test the a priori hypothesis that gene abundance would be higher in winter than in summer, as winter conditions (lower temperatures) would be expected to impose greater metabolic demands on the host and thus potentially drive enhanced microbial butyrate production. For transcriptome analyses, multiple testing was corrected using the Benjamini–Hochberg false discovery rate (FDR) method, with significance defined as FDR < 0.05. Correlations among serum butyrate concentrations, mRNA expression levels of *SLC5A8* and *SLC16A1*, and thermogenesis-related genes in adipose tissue were analyzed using two-sided Spearman’s rank correlation. A *p*-value < 0.05 was regarded as statistically significant. In the seasonal experiment, the experimental unit for metagenomic sequencing was each pooled fecal sample (composite of 4 pigs), with 3 pools per season. For all other measurements including serum and fecal butyrate concentrations and adipose transcriptomics, the experimental unit was the individual pig. The metagenomic sequencing data, together with the butyrate and transcriptomic data newly generated in the present study, were all derived from the same cohort of 12 Min pigs. In the acute cold challenge experiment, all analyses were performed with individual piglets as the experimental unit (*n* = 3 per group). All replicates reported are biological replicates.

## 3. Results

### 3.1. Long-Term Cold Exposure Reshapes the Gut Microbiota of Min Pigs and Enhances Butyrate Synthesis Potential

**Figure 1:**
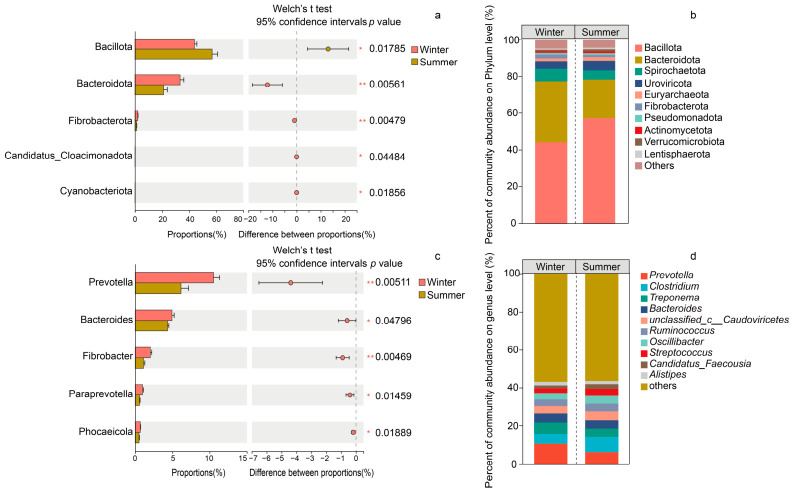
Seasonal differences in the gut microbiota composition of Min pigs between winter and summer. (**a**) Differential analysis at the phylum level; (**b**) Stacked bar plot illustrating the relative abundance of dominant bacterial phyla; (**c**) Differential analysis at the genus level; (**d**) Stacked bar plot illustrating the relative abundance of dominant bacterial genera. * *p* < 0.05, ** *p* < 0.01.

We first compared gut microbial composition between summer and winter to evaluate seasonal cold exposure effects on community structure. At the phylum level, the relative abundances of Fibrobacterota (*p* = 0.005), Bacteroidota (*p* = 0.006), Cyanobacterota (*p* = 0.019), and Candidatus Cloacimonadota (*p* = 0.045) were significantly enriched in winter compared with summer. Among these, Bacteroidota was dominant in both seasons and further enriched in winter (Figure 1a,b). At the genus level, the relative abundances of *Prevotella* (*p* = 0.005), *Fibrobacter* (*p* = 0.005), *Paraprevotella* (*p* = 0.015), *Phocaeicola* (*p* = 0.019), and *Bacteroides* (*p* = 0.048) were significantly enriched in winter (Figure 1c,d). Except for *Fibrobacter*, the other four genera with significantly increased abundance in winter all belong to the phylum Bacteroidota.

**Figure 2:**
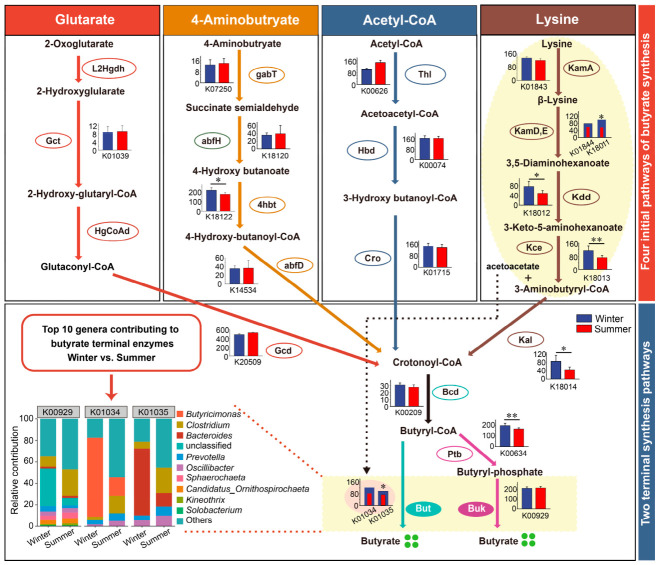
Seasonal variation in butyrate synthesis pathways and key enzyme gene abundance in Min pigs. Abundance of key genes in four initiation pathways (glutarate, 4-aminobutyrate, acetyl-CoA, and lysine) and two terminal pathways (but and buk). The lower left bar chart shows the relative contribution of the top 10 genera to terminal enzyme genes (K00929, K01034, K01035). * *p* < 0.05, ** *p* < 0.01.

Following the differential analysis of microbial composition, we subsequently compared the abundance profiles of butyrate synthesis-related functional genes. The key genes involved in the acetyl-CoA, glutarate, and 4-aminobutyrate pathways showed no significant seasonal differences between winter and summer (Figure 2). In contrast, several core genes of the lysine pathway (K18012, K18013, K18014) were significantly enriched in winter (Figure 2 and Figure S1 and Table S1). For the terminal pathways, genes of the but pathway (K00209, K01034, K01035) exhibited an upward trend in winter without reaching statistical significance. Within the buk pathway, the abundance of K00634 was significantly elevated (*p* < 0.01), whereas the terminal enzyme gene K00929 remained unchanged across seasons (Figure 2 and Figure S1 and Table S1).

To evaluate the relative contribution of each butyrate synthesis pathway, we normalized the abundances of key genes within each sample. The normalized abundance ratio of several functional genes were markedly elevated in winter, including K18011–K18014 in the lysine pathway, K18122 in the 4-aminobutyrate pathway, K01035 in the but terminal pathway, and K00634 in the buk pathway (Figure S2 and Table S2). By contrast, the normalized abundance of K00929, a terminal enzyme encoded by the buk pathway, remained unchanged. Collectively, these results indicate that enhanced butyrate synthesis under cold exposure did not arise from synchronous upregulation of all pathways, but mainly reflected selective enrichment of the lysine pathway.

We further analyzed the taxonomic contribution of bacteria to the terminal pathway genes K00929, K01034, and K01035, focusing on the top ten dominant genera. At the genus level, with the exception of *Butyricimonas*, all other nine genera contributed to K00929. *Clostridium*, *Prevotella*, and *Oscillibacter* contributed to both buk and but terminal enzymes, whereas *Bacteroides* contributed only to K01035, and *Butyricimonas* contributed exclusively to K01034. Taxonomically, *Clostridium* and *Oscillibacter* belong to Bacillota, while *Prevotella*, *Bacteroides*, and *Butyricimonas* belong to Bacteroidota. Notably, in winter, the functional contribution of K01034 was derived primarily from *Butyricimonas*, and that of K01035 mainly from *Bacteroides*, with the relative contribution of both genes being higher in winter than in summer (Figure 2). These results suggest that low temperature not only reshapes butyrate synthesis pathways, but also alters the microbial source composition of terminal enzyme genes.

### 3.2. Elevated Butyrate Levels Are Associated with Activation of Thermogenic Gene Expression in Adipose Tissue

**Figure 3:**
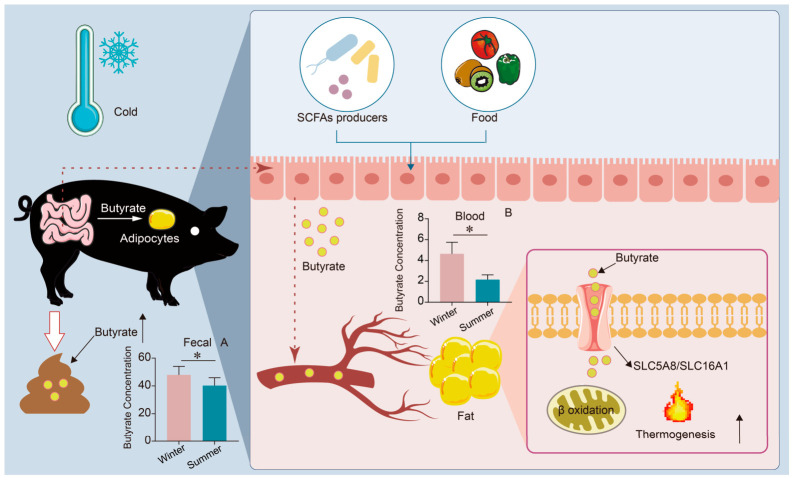
Seasonal differences in fecal and serum butyrate concentrations in Min pigs. Butyrate levels in feces (**A**) and serum (**B**) were significantly elevated in winter compared with summer. Arrows depict the complete metabolic route: gut-derived butyrate flows into feces and blood, is transported into adipocytes, and ultimately promotes thermogenesis (upward arrow). All data are presented as mean ± SD. * *p* < 0.05.

Fecal butyrate concentrations were significantly higher in winter than in summer in Min pigs (47.96 vs. 40.24 μmol/L, *p* = 0.020; Figure 3A). Similarly, serum butyrate levels were markedly elevated in winter (4.64 vs. 2.17 μmol/L, *p* = 0.046; Figure 3B).

**Figure 4:**
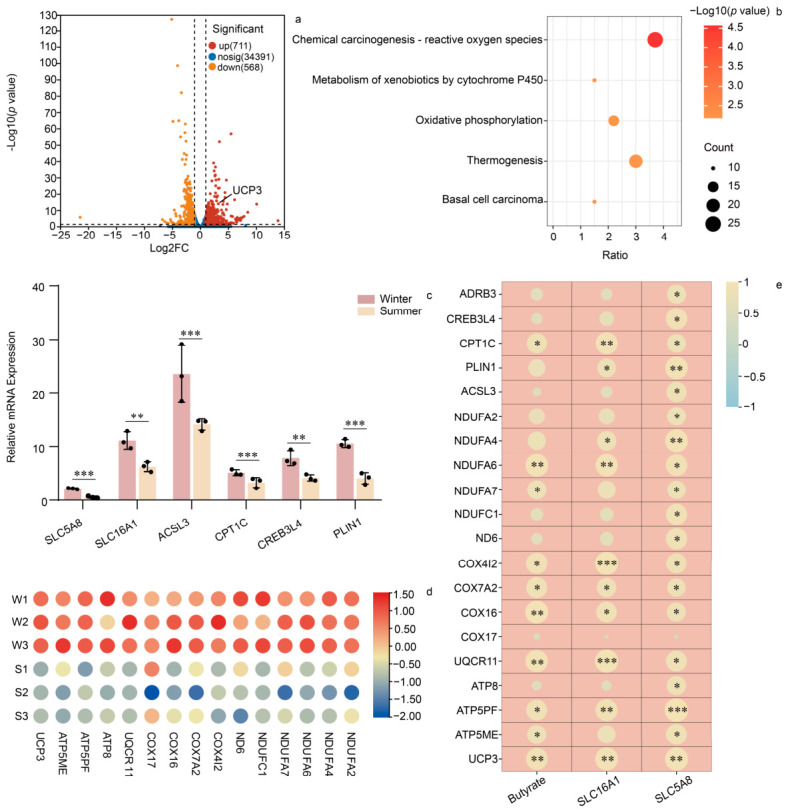
Seasonal changes in butyrate-related and thermogenic gene expression in subcutaneous adipose tissue of Min pigs, and correlations of serum butyrate and butyrate transporter genes in adipose with thermogenesis-related genes. (**a**) Volcano plot: red, orange and blue dots indicate genes upregulated, downregulated and unchanged in winter, respectively; (**b**) KEGG enrichment of winter-upregulated genes (bubble plot); (**c**,**d**) mRNA levels of genes involved in butyrate transport, thermogenesis, and oxidative phosphorylation; (**e**) Spearman correlation heatmap between serum butyrate, butyrate transporter gene expression and thermogenesis-related genes in adipose tissue. * *p* < 0.05, ** *p* < 0.01, *** *p* < 0.001.

Transcriptome analysis of subcutaneous adipose tissue identified 711 significantly upregulated and 568 downregulated genes in winter (|log_2_FC| ≥ 2, *p* < 0.05; Figure 4a). KEGG enrichment analysis showed that the upregulated genes were primarily enriched in pathways related to oxidative stress response, fatty acid metabolism, oxidative phosphorylation, and thermogenesis (Figure 4b). Among thermogenesis-related genes, the β-adrenergic receptor *ADRB3*, genes involved in β-oxidation, the mitochondrial uncoupling protein UCP3, along with genes associated with the electron transport chain and ATP synthesis complex, were all significantly upregulated in winter (adjusted *p* < 0.05; Figure 4c,d and Table S3). Additionally, the butyrate transporter gene *SLC5A8* (adjusted *p* = 2.64 × 10^−9^) and *SLC16A1* (adjusted *p* = 0.001) were also significantly upregulated (Figure 4c and Table S3).

Correlation analysis showed that both serum butyrate concentration and the expression of *SLC16A1* in subcutaneous adipose tissue were significantly positively correlated with 10 out of 20 thermogenesis-related genes (Figure 4e and Table S4). The expression of *SLC5A8* was significantly positively correlated with 19 of these thermogenesis-related genes (Figure 4e and Table S4). Collectively, these findings reveal that winter-induced increases in circulating butyrate and adipose butyrate transporter expression show strong positive correlations with the upregulation of thermogenesis-associated transcripts in subcutaneous adipose tissue.

### 3.3. Min Pigs Exhibit Greater Butyrate Synthesis Potential in Gut Microbiota than Large White Pigs

**Figure 5:**
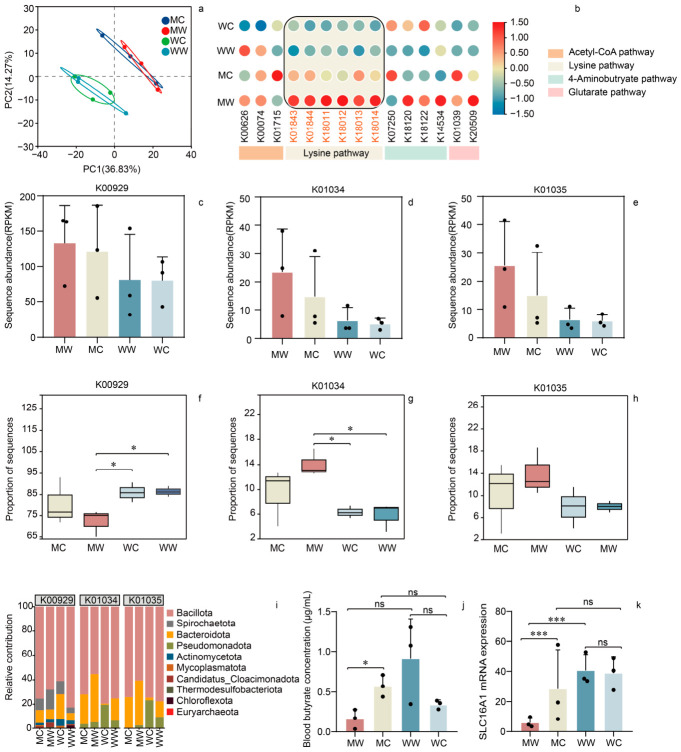
Breed comparison of microbial butyrate synthesis and host responses under cold stimulation. MC/MW: cold/warm Min pigs; WC/WW: cold/warm Large White pigs. (**a**) PCA of bacterial communities based on terminal butyrate synthesis genes (K00929, K01034, K01035); (**b**) Relative abundance of key genes in the four initiation pathways; (**c**–**e**) Abundance of terminal synthesis genes; (**f**–**h**) Relative contribution ratios of *but* versus *buk* terminal genes; (**i**) Phylum-level bacterial functional contributions to terminal synthesis genes; (**j**) Serum butyrate concentration; (**k**) mRNA levels of SLC16A1 in adipose tissue. * *p* < 0.05, *** *p* < 0.001, ns, not significant.

PCA revealed clear structural separation of the butyrate-producing microbiota between Min pigs and Large White pigs, indicating distinct gut microbial compositional profiles in the two breeds (Figure 5a). Comparison of gene abundances across the four butyrate synthesis pathways showed that, regardless of temperature conditions, six key genes involved in the lysine pathway (K01843, K01844, K18011–K18014) exhibited higher average abundance in Min pigs than in Large White pigs (Figure 5b). Moreover, the abundance in Min pigs under cold stress remained higher than that in Large White pigs under normal temperature conditions. Although these differences did not reach statistical significance due to individual variation, the overall trends were consistent with our seasonal observations, further supporting a pivotal role of the lysine pathway in mediating microbial butyrate synthesis in Min pigs.

We further compared the abundances of key genes involved in the two terminal pathways (buk and but) across pig breeds and temperature conditions. Under both thermal conditions, Min pigs exhibited higher abundances of these three terminal genes relative to Large White pigs (Figure 5c–e). We next evaluated the normalized abundance ratio of the buk and but pathways using the gene sets K00929, K01034, and K01035. The normalized abundance ratio of K00929 in thermoneutral Min pigs was significantly lower than that in Large White pigs under both thermoneutral (*p* = 0.045) and cold conditions (*p* = 0.043; Figure 5f). In contrast, the two key genes of the but pathway (K01034 and K01035) showed greater relative ratio in Min pigs at both temperatures (Figure 5g,h). Notably, the abundance ratio of K01034 in thermoneutral Min pigs was significantly higher than in Large White pigs under either thermoneutral (*p* = 0.010) or cold exposure (*p* = 0.013; Figure 5g).

Taxonomic contribution analysis further revealed that, for the but pathway, Bacteroidota dominated the contributions to K01034 and K01035, with this pattern being more pronounced in Min pigs (Figure 5i). Collectively, these findings highlight that Bacteroidota-derived taxa may provide an important functional contribution to microbial butyrate synthesis in the gut of Min pigs, with such functional contribution further strengthened under cold stress.

### 3.4. Cold Exposure Reveals Rapid Butyrate Metabolic Response in Min Pigs but Not in Large White Pigs

After acute cold challenge, serum butyrate concentrations were significantly increased in Min pigs (*t* = 3.93, *p* = 0.017; Figure 5j), accompanied by marked upregulation of the butyrate transporter gene *SLC16A1* in adipose tissue (adjusted *p* = 0.027; Figure 5k). In Large White pigs, however, neither serum butyrate levels (*t* = 1.95, *p* = 0.188) nor the expression of *SLC16A1* showed significant alterations following cold exposure (adjusted *p* = 1.000; Figure 5j,k).

Breed comparison revealed that under thermoneutral conditions, serum butyrate concentrations tended to be lower in Min pigs than in Large White pigs (*t* = 2.52, *p* = 0.115), while the expression of *SLC16A1* was significantly downregulated in Min pigs (adjusted *p* = 7.59 × 10^−12^). After cold challenge, serum butyrate levels tended to be higher in Min pigs relative to Large White pigs, although this difference did not reach statistical significance (*t* = 2.72, *p* = 0.083), whereas the breed difference in *SLC16A1* expression diminished to non-significant (adjusted *p* = 0.500; Figure 5j,k). For the alternative butyrate transporter *SLC5A8,* expression levels were extremely low in most samples, and thus this gene was not considered in the present analysis. Collectively, these findings indicate that Min pigs possess a rapid inducible butyrate metabolic response under acute cold stress, whereas Large White pigs lack this acute regulatory capacity. This highlights a distinct adaptive feature in Min pigs, with a low baseline butyrate and transporter profile that may allow stronger physiological upregulation upon cold exposure.

### 3.5. Metagenomic Binning Reveals Enrichment of Dual-Pathway and Dual-Terminal Butyrate-Producing Bacteria in Min Pigs

**Figure 6:**
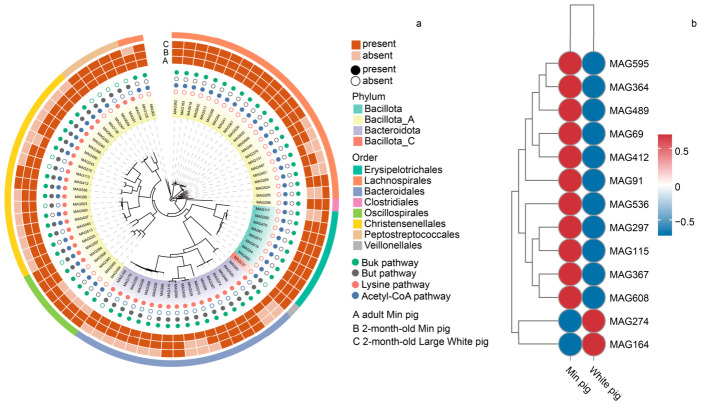
Phylogeny and pathway profiles of butyrate-producing bacterial MAGs from Min pigs and Large White pigs. (**a**) Phylogenetic tree of butyrate-synthesizing MAGs; colors and symbols denote the acetyl-CoA, lysine, and terminal but/buk pathways. The outer ring indicates sample origin: A, adult Min pigs; B, 2-month-old Min pigs; C, 2-month-old Large White pigs; (**b**) Relative abundance differences in lysine pathway-containing MAGs between the two breeds.

A total of 650 MAGs were recovered via metagenomic binning (Table S5), among which 78 encoded complete butyrate synthesis pathways. These MAGs were primarily affiliated with Bacillota, Bacillota_A, Bacillota_C, and Bacteroidota (Table S6). Pathway profiling showed that Bacillota, Bacillota_C, and nearly half of the Bacillota_A MAGs harbored only the acetyl-CoA pathway, whereas all Bacteroidota and some Bacillota_A MAGs possessed the lysine pathway. Notably, Bacteroidota MAGs relied exclusively on the lysine pathway for butyrate synthesis (Figure 6a). In total, 40 MAGs contained the full lysine pathway, of which 13 were shared by both pig breeds and the remaining 27 were uniquely detected in Min pigs, indicating distinct breed-specific distribution (Figure 6a and Table S7). The 13 shared lysine-pathway MAGs exhibited higher overall relative abundance in Min pigs than in Large White pigs (Figure 6b).

Terminal pathway analysis showed that most Bacteroidota MAGs carrying the lysine pathway possessed both but and buk terminal pathways, with only two MAGs lacking one pathway. By contrast, MAGs carrying only the acetyl-CoA pathway predominantly possessed only the buk pathway (Figure 6a). Notably, 15 MAGs belonging to Bacillota_A were identified that simultaneously harbored the lysine and acetyl-CoA pathways, along with both but and buk terminal pathways. Thirteen of these dual-pathway MAGs were assigned to the order Christensenellales (family CAG-138) and two to Oscillospirales (family Oscillospiraceae), with genera including *PeH17*, *SFDB01*, *RGIG1693*, and *Vescimonas*. Of the 15, 11 were exclusive to Min pigs (Figure 6a). Enrichment of these dual-pathway, dual-terminal strains may enhance metabolic redundancy and functional robustness in the gut microbiota of Min pigs, providing a critical microbial foundation for stable energy supply under cold stress.

## 4. Discussion

By integrating long-term seasonal cold exposure and acute cold challenge, we systematically characterized the adaptive regulation of the “cold–gut microbiota–butyrate metabolism–adipose thermogenesis” axis in Min pigs across microbial composition, butyrate synthesis pathways, metabolite profiles, and adipose transcriptional responses. Prolonged cold exposure markedly reshaped gut microbial functional capacity and host energy metabolism, with selective enrichment of the lysine-dependent butyrate synthesis pathway during chronic cold adaptation.

Butyrate concentrations in Min pigs increased markedly under both long-term cold acclimation and acute cold challenge, consistent with previous findings in sheep [42,43]. These findings further support a conserved regulatory role for butyrate in mammalian cold adaptation. Combined with metagenomic functional analyses, our results indicate that the cold-associated increase in butyrate was not driven by a synchronous enhancement of all synthetic pathways, but rather by selective remodeling of specific pathways. Specifically, key genes of the lysine pathway were universally upregulated in winter, whereas the acetyl-CoA, glutarate, and 4-aminobutyrate pathways remained largely stable. At the terminal reaction level, the relative ratio of the but pathway increased. For the buk pathway, although upstream related genes K00634 were elevated in winter, the terminal enzyme K00929 showed no significant change. Given that terminal enzymes are reliable indicators of microbial butyrate production capacity, these findings indicate that the enhanced butyrate synthesis in the gut microbiota of cold-exposed Min pigs relies on coordinated functional shifts toward the lysine pathway and the but terminal pathway, rather than generalized upregulation across all synthetic pathways.

This pathway shift likely reflects an underlying metabolic rationale. A potential biochemical coupling exists between the lysine pathway and the but pathway. Acetoacetate generated via the lysine pathway can serve as a cosubstrate acceptor for the but reaction, together with butyryl-CoA, to generate butyrate and acetyl-CoA. The latter can then re-enter the mainstream metabolic pathway and continue to participate in butyryl-CoA synthesis [13,44]. By contrast, if lysine-derived butyryl-CoA is preferentially directed toward the buk pathway, the accompanying acetoacetate may be less efficiently reutilized, thereby reducing overall metabolic economy. Accordingly, the coordinated enrichment of the lysine and but pathways indicates that cold-exposed gut microbiota adapt to host demand not only by boosting butyrate flux, but also by optimizing substrate coupling and terminal pathway coordination to enhance butyrate production efficiency.

This metabolic strategy relies on enrichment of specialized functional taxa. Our results showed that Bacteroidota was significantly enriched in the gut of Min pigs during winter, which is consistent with previous findings [19], and also agrees with reports of increased Bacteroidota abundance during cold seasons in Tibetan sheep, wild pikas, yaks, and brown bears [45]. Previous studies have shown that Bacteroidota is a key contributor to the lysine-dependent butyrate synthesis pathway, with genomes often harboring relatively complete sets of related pathway genes [46]. Our metagenomic binning results further revealed that butyrate-synthesizing MAGs derived from Bacteroidota generally possessed both the lysine pathway and dual terminal pathways.

Taken together, these findings suggest that the regulation of butyrate synthesis in Min pigs under cold exposure may involve not simply an increase in the total abundance of butyrate-producing bacteria, but rather the enrichment of key functional taxa with advantageous pathway configurations, thereby optimizing substrate utilization and terminal reaction patterns to improve butyrate synthesis efficiency and provide more stable metabolic support for host cold adaptation.

Beyond microbial remodeling, multiple thermogenesis-related genes were significantly upregulated in the adipose tissue of Min pigs in winter. Notably, the expression levels of the butyrate transporter genes *SLC5A8* and *SLC16A1* were markedly increased and showed strong positive correlations with core thermogenic genes. Consistent with these observations, the cold-triggered rise in circulating butyrate was accompanied by upregulated expression of thermogenesis-related genes in subcutaneous adipose tissue, implying a potential molecular link between gut butyrate and adipose transcriptional responses under cold exposure.

Previous studies have confirmed that butyrate promotes thermogenesis in brown adipose tissue by upregulating UCP1 expression [12]. Recent evidence further indicates that butyrate enters host cells through MCT1, encoded by *SLC16A1*, and activates the epigenetic regulator LSD1 to induce expression of UCP1 and other thermogenesis-related genes [11]. Although pigs lack functional *UCP1*, winter exposure significantly upregulated *UCP3*, a paralog contributing to porcine thermogenesis [47], alongside genes involved in fatty acid oxidation and the electron transport chain. Correlation analyses confirmed that serum butyrate, *SLC5A8* and *SLC16A1* expression were strongly correlated with most thermogenic genes. This suggests that circulating butyrate may be involved in cold adaptation via UCP3-mediated uncoupling and enhanced mitochondrial oxidative metabolism. Elevated butyrate may be transported into adipose tissue via specific carriers, potentially contributing to the transcriptional regulation of thermogenic gene programs. However, these interpretations should be viewed with caution given several limitations. The correlation analyses were based on a small sample size (*n* = 3 per season), limiting their statistical reliability. Thus, they should be considered exploratory and warrant independent validation in larger cohorts. The 3-day cold challenge was designed to capture acute transcriptional responses rather than chronic structural remodeling of adipose tissue, and the absence of histomorphological data (e.g., adipocyte size, mitochondrial density, or UCP1 protein levels) further constrains our ability to assess browning-like changes. Accordingly, our conclusions regarding adipose tissue adaptation are confined to the transcriptional level, and longer-term studies incorporating histological and functional analyses are required to determine whether sustained cold exposure induces structural and functional remodeling.

Breed comparisons suggested that the gut microbiota of Min pigs showed higher gene abundance for butyrate synthesis than that of Large White pigs. Under thermoneutral conditions, Min pigs already exhibited higher baseline abundance of genes related to terminal butyrate synthesis, with prominent enrichment of the key but pathway gene K01034. Among the four initial synthesis pathways, genes involved in the lysine pathway were also consistently enriched in Min pigs. Combined with the seasonal upregulation of both the lysine pathway and the but pathway in winter, these findings suggest that Min pigs maintain higher basal gene abundance for microbial butyrate production, with further shifts in gene abundance toward these pathways under cold stress. This microbial feature may be associated with the cold adaptation of Min pigs.

Metagenomic binning further supported this interpretation. We identified 40 MAGs carrying the lysine-dependent butyrate synthesis pathway, 27 of which were exclusive to Min pigs, while the remaining 13, though shared, were generally more abundant in Min pigs. Given that Bacteroidota is an important carrier of the lysine pathway, this result also explains at the genomic level why Min pigs exhibit significantly greater functional potential in this pathway than Large White pigs. Notably, 15 dual-pathway and dual-terminal MAGs were identified, harbouring both the lysine and acetyl-CoA pathways alongside dual but and buk terminal systems. Among these 15 MAGs, 11 were detected only in Min pigs. Such genomic configurations suggest that the corresponding microbial populations may possess greater metabolic redundancy and environmental adaptability, potentially allowing them to utilize alternative butyrate synthesis routes depending on substrate availability. However, these inferences are based on genomic potential rather than direct measurements of pathway activity or metabolic flux, and functional validation is required to confirm their contributions to butyrate production in vivo.

Overall, cold adaptation in Min pigs relies on coordinated crosstalk between host metabolism and gut microbiota, integrating microbial functional remodeling, enhanced butyrate production, and transcriptional activation of thermogenic genes. Under cold stress, the gut microbiota of Min pigs optimizes butyrate synthesis by selectively enriching the lysine-but pathway. Meanwhile, the host upregulates short-chain fatty acid transporters and thermogenic programs, potentially supporting butyrate uptake and downstream metabolic utilization. By contrast, Large White pigs showed relatively limited changes in butyrate levels and transporter-associated responses after cold challenge, suggesting weaker microbial metabolic reserve, pathway flexibility, and host utilization capacity than Min pigs. These findings provide a theoretical basis for targeting gut microbial butyrate synthesis pathways via nutritional strategies to improve cold adaptation and feed efficiency in agricultural animals.

Nevertheless, several limitations should be acknowledged. The use of different age groups introduces age as a potential confounding factor, as physiological status, gut microbial community structure, and adipose thermogenic capacity differ substantially between adults and juveniles. In addition, the seasonal study used fecal samples whereas the acute challenge used cecal contents, which differ in microbial composition and are not directly quantitatively comparable. Thus, the two experiments are complementary rather than directly comparable, with the seasonal study reflecting long-term chronic adaptation in adults and the acute challenge capturing rapid breed-specific responses in young animals. Direct cross-age comparisons are therefore avoided. Future studies should test whether the observed pathway shifts are reproducible in adult pigs under acute cold and in young piglets under chronic cold. Beyond these design limitations, the small sample size limits statistical power, though the observed differences were consistent across multiple independent measurements. All results require validation in larger cohorts and are presented as exploratory. Most importantly, our study is largely correlational. Causality requires direct interventions such as fecal microbiota transplantation or butyrate supplementation. We also acknowledge that seasonal metagenomic data were derived from pooled fecal samples, so metagenome-host correlations are exploratory. However, serum butyrate-transcriptome correlations are based on paired individual data, and all multi-omics data in the acute experiment were fully paired. The consistency of key findings across both experiments supports the robustness of our conclusions despite these limitations.

## 5. Conclusions

Our findings suggest that long-term cold exposure was associated with enrichment of the lysine-but pathway and increased contribution of Bacteroidota-derived taxa to microbial butyrate synthesis in Min pigs. Breed comparisons indicated that Min pigs have a higher lysine-but pathway abundance for butyrate synthesis than Large White pigs. Metagenomic binning revealed that MAGs harboring both lysine and acetyl-CoA pathways, as well as dual terminal but and buk pathways for butyrate synthesis, were predominantly found in Min pigs. Bacteroidota-derived butyrate-producing MAGs in our dataset carried the lysine pathway along with both but and buk terminal pathways, and were more widely distributed in Min pigs than in Large White pigs. These findings suggest that dietary strategies targeting the lysine-but pathway (e.g., lysine supplementation or Bacteroidota-promoting prebiotics) and improved housing management could be explored to enhance cold tolerance in pigs raised in cold regions such as Heilongjiang, providing a theoretical foundation for region-specific nutritional and management interventions in northern farming systems.

## Data Availability

Raw sequencing data generated from seasonal subcutaneous adipose transcriptome of Min pigs, adipose transcriptome of Min pigs and Large White pigs under acute cold challenge, and cecal metagenome of the two breeds under control and cold conditions were deposited in the NCBI SRA database under accession numbers PRJNA1414131, PRJNA1414151, and PRJNA1414139, respectively.

## Supplementary Materials

The following supporting information can be downloaded at: https://www.mdpi.com/article/10.3390/microorganisms14071575/s1. Figure S1: Seasonal comparison of butyrate synthesis gene abundance in the gut microbiota of Min pigs; Figure S2: Seasonal comparison of relative contributions of butyrate synthesis genes in Min pigs; Table S1: Statistical results of Welch’s *t*-test for seasonal differences in RPKM abundance of butyrate synthesis genes in the gut microbiota of Min pigs; Table S2: Statistical results of Welch’s *t*-test for seasonal differences in relative contributions of butyrate synthesis genes in the gut microbiota of Min pigs; Table S3: Statistical summary of thermogenesis-related genes upregulated in winter compared with summer; Table S4: Correlation analysis among serum butyrate concentration, butyrate transporter gene expression in adipose tissue and thermogenesis-related gene expression; Table S5: Basic information of 650 MAGs; Table S6: The taxonomic classification and abundance information of 78 butyrate-producing bacteria; Table S7: The taxonomic classification and abundance information of 40 butyrate-producing bacteria harboring the lysine pathway.

## Abbreviations

The following abbreviations are used in this manuscript:

ELISA	Enzyme-linked immunosorbent assay
FC	Fold change
FDR	False discovery rate
GC-MS	Gas chromatography-mass spectrometry
SCFAs	Short-chain fatty acids
