# Supplementary material for: Cold Exposure Shifts Gut Microbial Butyrate Synthesis Toward the Lysine-Dependent and But-Mediated Terminal Pathways to Enhance Cold Tolerance in Min Pigs
Source: Microorganisms. 2026 Jul 19;14(7):1575. doi: 10.3390/microorganisms14071575 (PMC13413642; doi:10.3390/microorganisms14071575)
Supplement: Supplementary file 1 [file microorganisms-14-01575-s001.zip › Supplemental Information.pdf]

Supplementary Information

Cold Exposure Shifts Gut Microbial Butyrate Synthesis Toward the Lysine-but Pathway to Enhance Adipose Thermogenesis and Cold Tolerance in Min Pigs

Yang Chang [1], Xinlei Liu [1], Lujing Song [1], Fei Xu [1], Ziwen Zhang [1], Miao Yu [1], Guandong Wu [1], Dongjie Zhang [2,*] and Chunzhu Xu [1,*]

[1] Key Laboratory of Animal Cellular and Genetics Engineering of Heilongjiang Province, College of Life Science, Northeast Agricultural University, Harbin 150030, China; changy051@neau.edu.cn (Y.C.); s2509012002@neau.edu.cn (X.L.); 18911250958@163.com (L.S.); s240901025@neau.edu.cn (F.X.); lisa_i_like@163.com (Z.Z.); s230901001@neau.edu.cn (M.Y.); s230902039@neau.edu.cn (G.W.)

[2] Institute of Animal Husbandry, Heilongjiang Academy of Agricultural Sciences, Harbin 150086, China

* Correspondence: zhangdongjie@haas.cn (D.Z.); cxu@neau.edu.cn (C.X.)

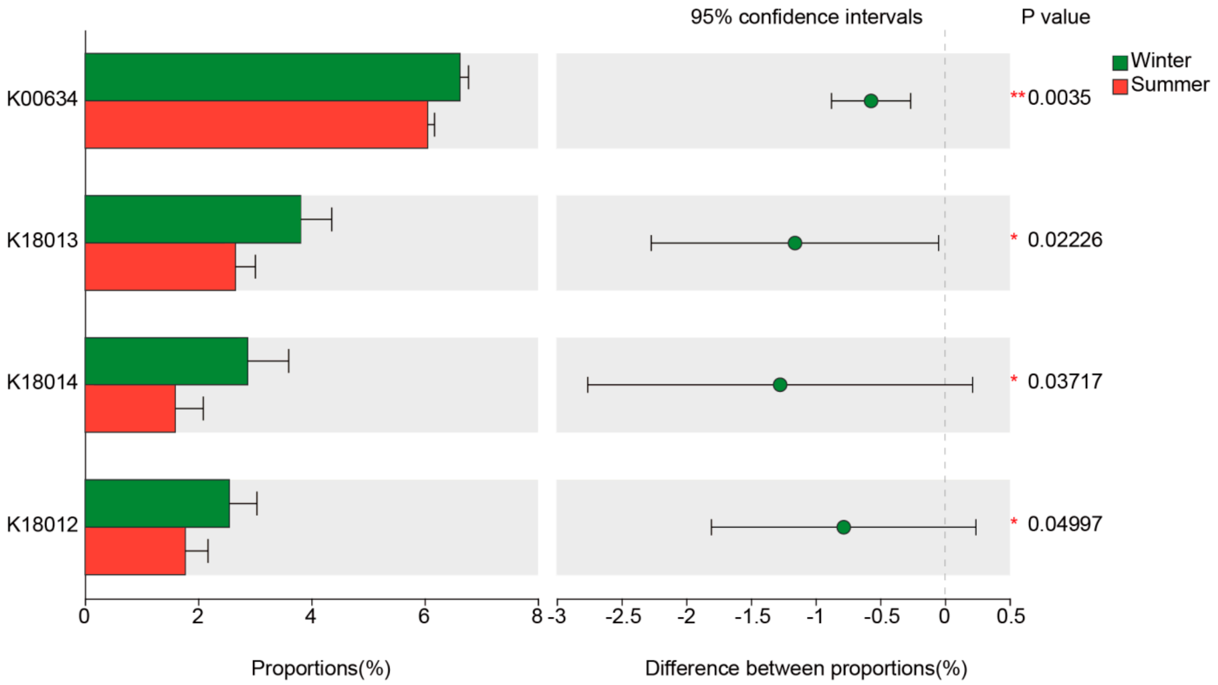

Figure S1. Seasonal comparison of butyrate synthesis gene abundance in the gut microbiota of Min pigs.

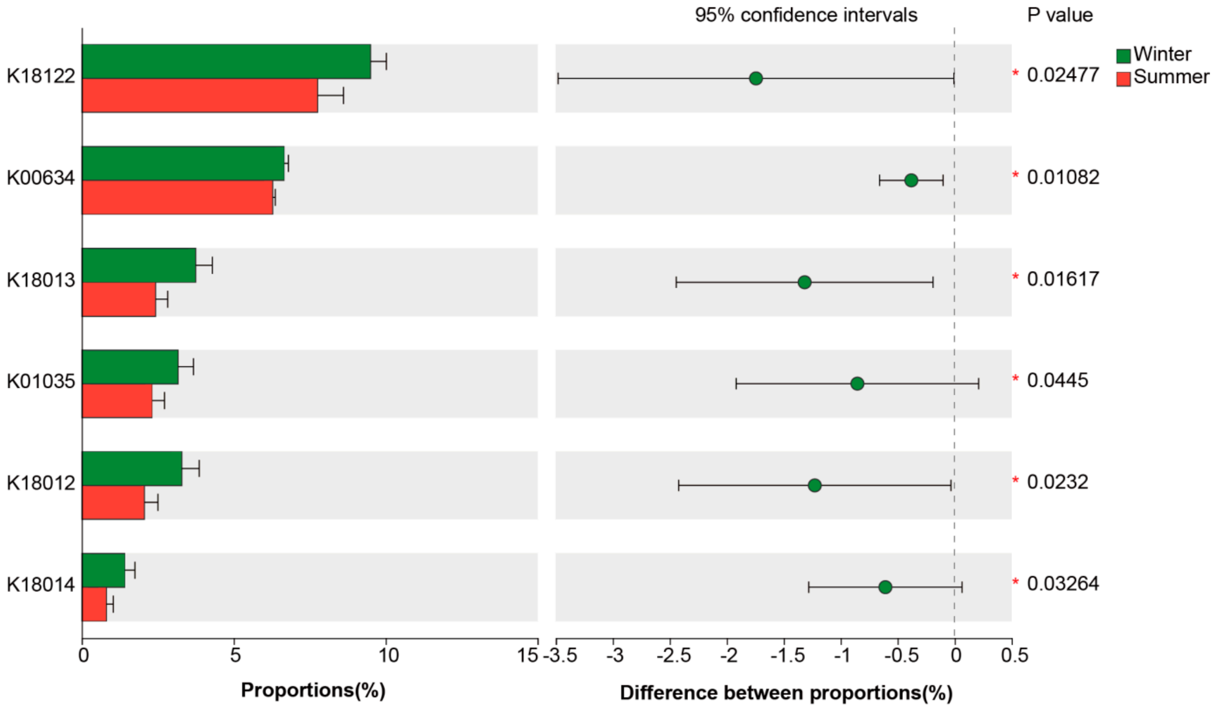

Figure S2. Seasonal comparison of relative contributions of butyrate synthesis genes in Min pigs.
